# Increased expression of heat shock factor 1 (HSF1) is associated with poor survival in gastric cancer patients

**DOI:** 10.1186/s13000-018-0755-3

**Published:** 2018-10-17

**Authors:** Weigang Dai, Jinning Ye, Zhimei Zhang, Liang Yang, Hui Ren, Hui Wu, Jianhui Chen, Jieyi Ma, Ertao Zhai, Shirong Cai, Yulong He

**Affiliations:** 1 Department of Gastrointestinal Surgery, First Affiliated Hospital, Sun Yat-sen University, 58 Zhongshan 2nd Road, Guangzhou, 510080 China; 2 Department of Pathology, First Affiliated Hospital, Sun Yat-sen University, Guangzhou, China; 3 Department of General Surgery, Seventh Affiliated Hospital, Sun Yat-sen University, Shenzhen, China; 4 General Surgical Laboratory, First Affiliated Hospital, Sun Yat-sen University, Guangzhou, China

**Keywords:** HSF1, Gastric cancer, Prognosis

## Abstract

**Background:**

Heat shock factor 1 (HSF1) was initially identified as a transcription factor encoding heat shock proteins, which assist in refolding or degrading damaged proteins. Recent studies have reported that HSF1 can act as an oncogene that regulates tumour progression. The present study aimed to elucidate the clinicopathological significance and prognostic value of HSF1 expression in gastric cancer (GC).

**Methods:**

The data from The Cancer Genome Atlas (TCGA) were used to analyse HSF1 expression in GC and normal tissues, while 8 pairs of freshly frozen tissue samples were used to investigate HSF1 expression at the mRNA and protein levels in GC tissues and adjacent normal tissues using quantitative real-time polymerase chain reaction (qRT-PCR) and western blotting assays. The correlations between HSF1 expression and clinicopathological parameters, including the survival rate, were investigated in 117 GC tissue samples by immunohistochemical analysis.

**Results:**

The results of bioinformatics analysis, qRT-PCR, and western blot showed that HSF1 expression was higher in GC tissues than in normal tissues. High HSF1 expression was found in 54.7% (64/117) patients. Patients with high HSF1 expression had larger tumour size (*P* = 0.001), advanced Bornmann classification (*P* = 0.002), advanced depth of invasion (*P* = 0.015), lymph node metastasis (*P<*0.001), distant metastasis (*P* = 0.011) and tumour-node-metastasis (*P<*0.001). Moreover, the Kaplan-Meier and Cox proportional hazards analyses indicated that high HSF1 expression was significantly associated with poor overall survival and recurrence-free survival in GC patients and that high HSF1 expression was an independent prognostic factor for the long-term survival in GC patients.

**Conclusions:**

Taken together, our results show that high HSF1 expression is significantly correlated with advanced tumour progression and poor prognosis. In addition, HSF1 expression can serve as a biomarker for the prognosis of patients with GC.

## Background

Gastric cancer (GC), one of the most common malignant tumour types, is the leading cause of deaths from cancer worldwide [1]. The incidence of gastric cancer is rising steadily in China [2]. Despite advances in surgery and chemotherapy, the prognosis of patients with advanced GC remains poor [3]. Therefore, it is of great significance to further understand the molecular mechanism controlling GC growth and progression and explore novel targeted drugs for improving the prognosis of GC patients.

Heat shock factor 1 (HSF1) was initially identified as a transcription factor upregulating genes that encode heat shock proteins (HSPs), which assist in refolding or degrading damaged proteins [4]. HSF1 can be primarily activated in response to heat stress, and its activation is accomplished at the protein-protein interaction and post-translational modification level [5]. In recent years, HSF1 has been revealed to modulate the endoplasmic reticulum unfolded protein response, oxidative stress response, autophagy, multidrug resistance, ubiquitin-proteasome system, chromatin remodelling, etc. [4, 6–11]. Moreover, HSF1 can regulate some cellular behaviours, such as apoptosis, proliferation, etc. [4].

Emerging evidence demonstrates that HSF1 is upregulated in human malignant tumours and acts as an oncogene to regulate tumour carcinogenesis and progression. HSF1 is overexpressed in several cancer types, including colorectal cancer (CRC), esophageal squamous cell carcinoma (ESCC), breast cancer (BC), hepatocellular carcinoma (HCC), osteosarcoma, non-small-cell lung cancer, clear cell renal cell carcinoma (ccRCC), etc. [12–18]. Moreover, a high HSF1 level in tumour tissues serves as a poor prognosis predictor in patients with carcinoma [12–19]. However, although it has been reported that HSF1 can downregulate ArgBP2 and regulate some cellular behaviours, such as apoptosis, proliferation, invasion and migration in GC [20–22], the clinicopathological significance and prognostic role of HSF1 expression in GC has not been understood. Therefore, in this study, we assessed the HSF1 expression in several GC specimens and investigated its association with clinicopathologic parameters, as well as with the long-term overall and recurrence-free survival of GC patients.

## Methods

### Patients and human tissue specimens

The files of 117 patients who had undergone surgical resection of GC at the First Affiliated Hospital of Sun Yat-sen University (FAHSYSU) between 2005 and 2006 were randomly chosen and analysed. Patients, who received neoadjuvant chemotherapy, lost to follow-up and cases with incomplete data, were excluded in this study. We reviewed the clinicopathological characteristics such as age, gender, tumour size, tumour location, tumour differentiation, histologic type, Bornmann classification, depth of invasion, lymph node metastasis, distant metastasis, tumour-node-metastasis stage and carcinoembryonic antigen (CEA) level. These cases were evaluated according to the 8th Edition of the American Joint Cancer Committee TNM classifications. The follow-ups were terminated by December 2017.

Fresh samples from resection specimens were collected from patients with primary GC who were treated by gastric surgery at FAHSYSU in 2017 (*N* = 8). These tissues were used to detect the mRNA and protein levels of HSF1 by qRT-PCR and western blotting.

Ethical approval for human subjects was obtained from the Institutional Review Board of the First Affiliated Hospital of Sun Yat-Sen University (FAHSYSU), and written consent was obtained from each patient.

### Immunohistochemical (IHC) staining

Paraffin-embedded GC tissues were obtained from the Department of Pathology and made into tissues microarrays for IHC staining. IHC staining was conducted as previously described [23]. The anti-HSF1 antibody (1:100, ab52757, Abcam, USA) was detected by IHC. We semi-quantitatively scored the expression levels of HSF1 according to the method used in our previous studies [24], with slight modifications. The extent of HSF1 staining was scored by assigning the percentage of positive tumour cells (0, none; 1, < 20% of positively staining cells; 2, 20–50% of positively staining cells; 3, > 50% of positively staining cells). Low HSF1 expression was referred to as the IHC score 0 and 1, and high HSF1 expression was referred to as the IHC score 3 and 2. Immunohistochemical evaluation was performed by two pathologists who were blind to the clinical and pathological characteristics associated with the specimens.

### Western blotting

Cell lysates were obtained as previously described [23]. Total protein was extracted with cell lysis buffer (KeyGene, Nanjing, China), and protein concentration was quantified using an Enhanced BCA Protein Assay Kit (KeyGene, Nanjing, China). The primary antibodies used were as follows: anti-HSF1 ((1:100, ab52757, Abcam, USA) and anti-GAPDH (Proteintech, Wuhan, China).

### Quantitative real-time polymerase chain reaction (qRT-PCR)

Total RNA and complementary DNA were isolated and prepared according to the protocol supplied with PrimeScript RT Reagent (TaKaRa, Japan) and PrimeScript RT Reagent (TaKaRa, Japan) according to the protocol supplied with the PrimeScript RT Reagent (TaKaRa, Japan). Expression of *HSF1* was determined by qRT-PCR using Power SYBR Green PCR master mix (Applied Biosystems). Oligonucleotide sequences of the primer sets used were as follows: *HSF1* (forward: 5’-TAATACGACTCACTATAGGG-3′, reverse: 5’-CTGGAATAGCTCAGAGGC-3′); *GAPDH* (forward: 5’-TGTGGGCATCAATGGATTTGG-3′ and reverse: 5’-ACACCATGTATTCCGGGTCAAT-3′).

### Bioinformatics analysis

The RNASeq data for GC were downloaded from The Cancer Genome Atlas (TCGA) databases (https://genome-cancer.ucsc.edu). The prognostic role of HSF1 expression was analysed in the Kaplan-Meier Plotter (http://kmplot.com). The data from TCGA were log_2_-transformed and analysed using the GraphPad Prism 5.0 software.

### Statistical analyses

The SPSS version 18.0 (IBM, USA) was used for data analysis. The relationship between the HSF1 expression and the features of tumour progression were analysed using the Chi-square tests. The Kaplan-Meier survival curves were constructed, and the log-rank test was carried out in univariate analysis. Multivariate analysis was performed using Cox’s proportional hazards model. A *P*-value of 0.05 was considered as statistically significant in all analyses.

## Results

### *HSF1* was highly expressed in GC tissues compared with adjacent normal tissues

**Fig. 1:**
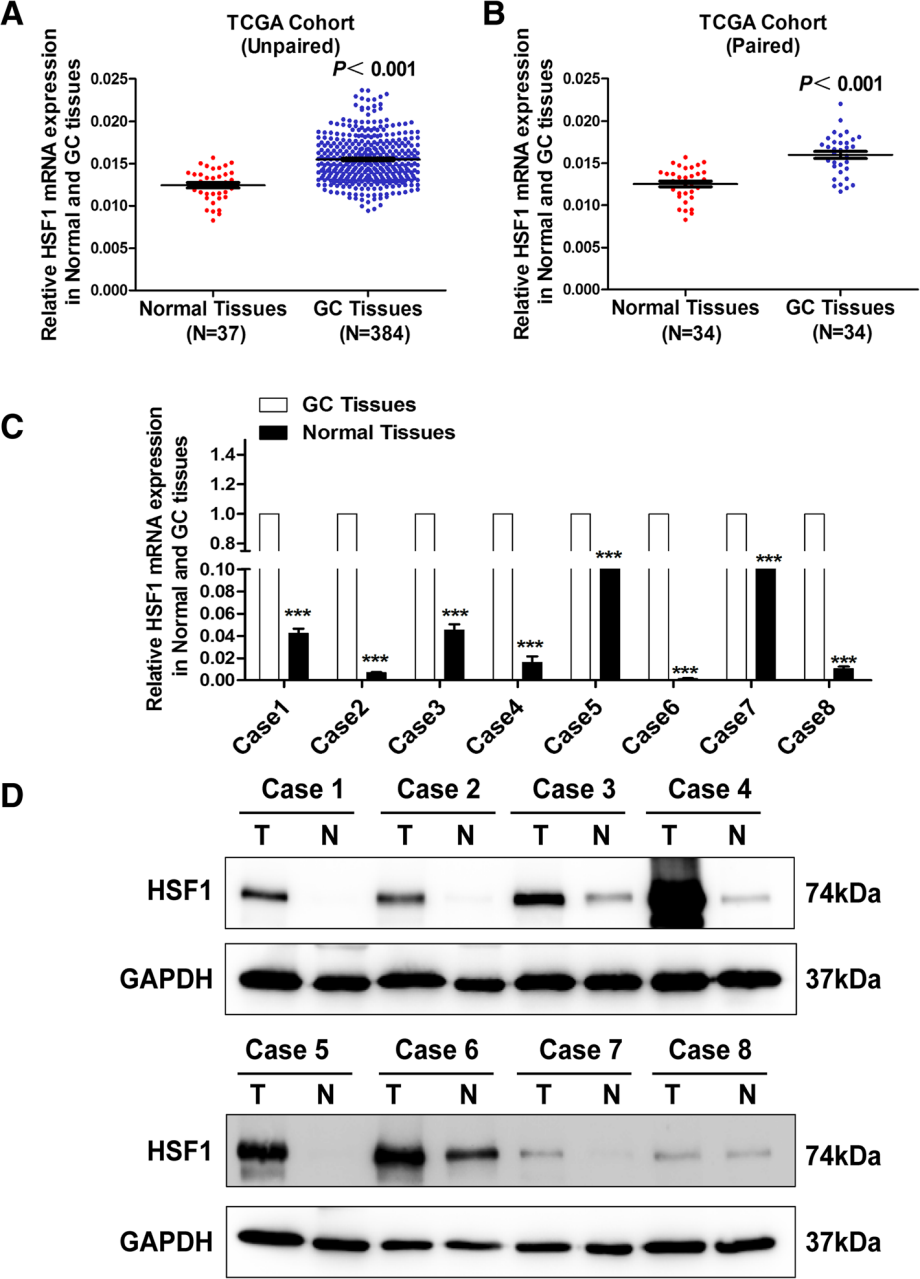
High *HSF1* expression in gastric cancer. (**a**) Analysis of *HSF1* expression in unpaired GC (*N* = 384) and normal tissues (*N* = 37) in the TCGA cohort (*P*<0.001); (**b**) *HSF1* expression in paired normal and GC tissues (*N* = 34) in the TCGA cohort (*P*<0.001); (**c**) Expression of *HSF1* was analysed by qRT-PCR in paired tumour and normal tissues; (**d**) HSF1 protein levels were measured in GC tissues and respective adjacent non-tumour tissues in 8 typical patients by western blotting

To evaluate *HSF1* expression in GC tissues, we first analysed *HSF1* mRNA expression in the TCGA cohort. The data indicated that *HSF1* mRNA level was markedly increased in the unpaired (Fig. 1a, *P* <0.001) and paired (Fig. 1b, *P* <0.001) GC tissues compared with normal tissues. We then verified *HSF1* mRNA and protein levels by qRT-PCR and western blotting assays in 8 pairs of fresh GC and normal tissues from our medical centre. We found that the *HSF1* mRNA (Fig. 1c) and protein (Fig. 1d) expression was increased in GC tissues compared to non-tumorous tissues. Collectively, these results indicated that *HSF1* is highly expressed in GC tissues compared with normal tissues.

### Associations of HSF1 expression with clinical parameters in GC

**Fig. 2:**
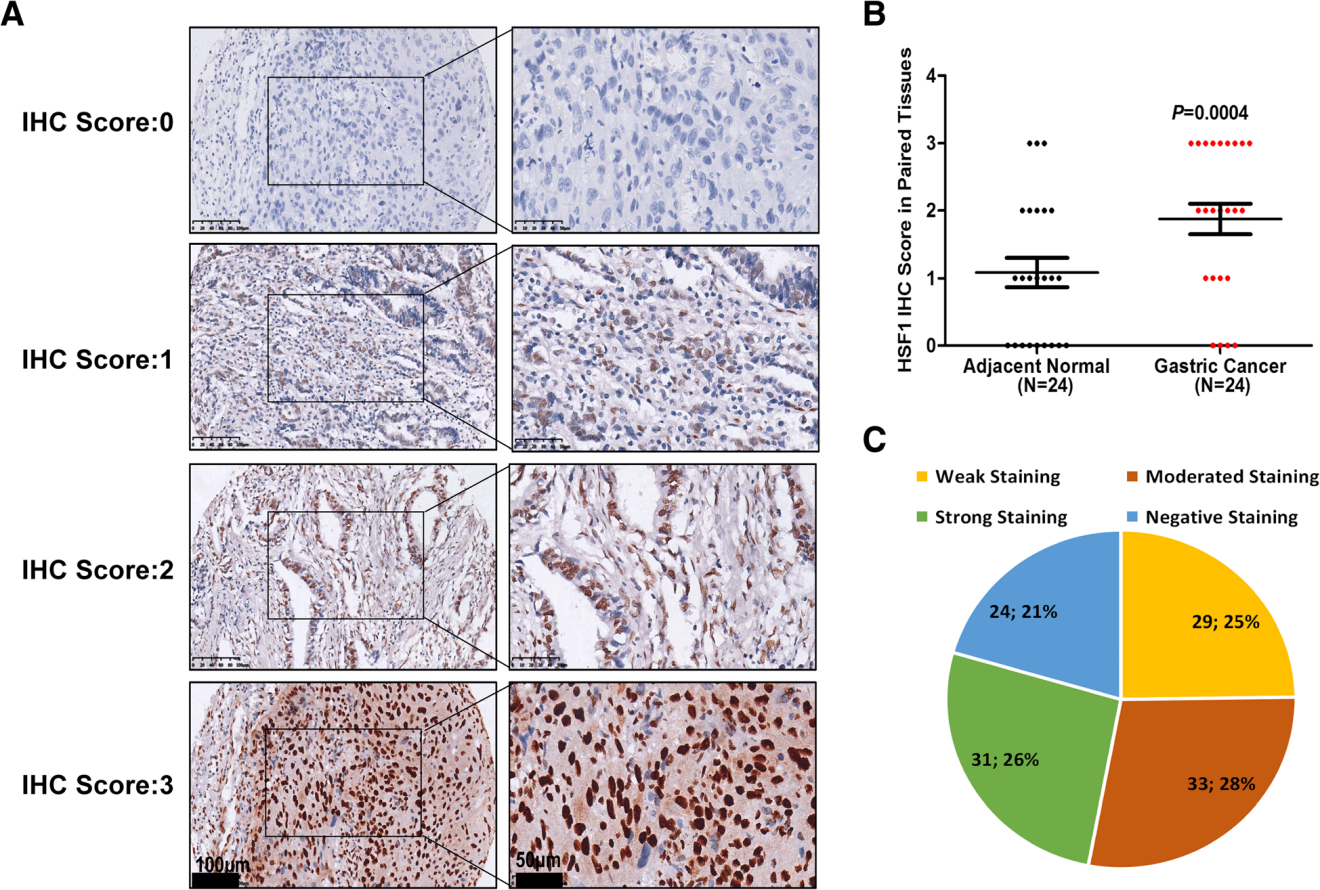
HSF1 protein expression in normal tissues and GC tissues. (**a**) IHC staining of HSF1 protein in GC tissues. IHC score was determined according to the percentage of positively staining cells (IHC score: 0, negative staining; IHC score: 1, < 20% of positively staining cells; IHC score: 2, 20–50% of positively staining cells; IHC score: 3, > 50% of positively staining cells); (**b**) HSF1 protein expression was significantly increased in primary tumour specimens compared with adjacent non-tumour tissues by IHC (*P* = 0.0004, *N* = 24); (**c**) Percentage of patients with GC according to the HSF1 protein expression by IHC scoring

To explore the associations between HSF1 expression and GC clinicopathologic characteristics, we performed immunohistochemistry to detect HSF1 expression in the GC tissue array, which contained 117 cases of GC tissue from GC patients. As shown in Fig. 2, HSF1 protein was mainly distributed in the nucleus (Fig. 2a). The protein expression of HSF1 was significantly higher in primary GC compared with adjacent normal tissues (Fig. 2b). As shown in Fig. 2c, there were 29 cases (25%) with IHC score 1, 33 cases (28%) with IHC score 2, 31 cases (26%) with IHC score 3, and 24 cases (21%) with negative staining (IHC score 0). Negative and weak staining were defined as low HSF1 expression (54.7%, 64/117), whereas moderate and strong staining were defined as high HSF1 expression (45.3%, 53/117).

The relationships between HSF1 expression and major clinical pathological features of GC were determined by the Chi-square test and are summarized in Table 1. The results indicated that high HSF1 expression in GC tissues was significantly correlated with larger tumour size (*P* = 0.001), advanced Bornmann classification (*P* = 0.002), advanced depth of invasion (*P* = 0.015), lymph node metastasis (*P*<0.001), distant metastasis (*P* = 0.011) and tumour-node-metastasis (*P*<0.001). However, there were no statistically significant relationships between HSF1 expression and other clinicopathological variables, such as gender (*P* = 0.459), age (*P* = 0.814), tumour location (*P* = 0.341), histological type (*P* = 0.483), differentiation (*P* = 0.225) and CEA (*P* = 0.709).

**Table 1:** Correlations between HSF1 expression and the clinicopathologic variables of 117 GC patients

Characteristic	N	HSF1 Expression		Χ^2^ Value	*P* value
		Low(*N* = 53)	High(*N* = 64)		
Gender					
Male	73	35	38	0.548	0.459
Female	44	18	26		
Age		56.87 ± 11.03	54.34 ± 13.821		
<60 y	72	32	40	0.055	0.814
≥ 60 y	45	21	24		
Tumor Location					
Proximal	34	15	19	3.35	0.341
Middle	33	12	21		
Distal	41	23	18		
More than 2	6	2	4		
Tumor size					
<5 cm	67	39	28	10.545	0.001
≥ 5 cm	50	14	36		
Histologic type					
Adenocarcinoma	98	43	55	0.492	0.483
others	19	10	9		
Bornmann classification					
1	6	3	3	14.657	0.002
2	24	17	7		
3	73	32	41		
4	14	1	13		
Differentiation					
Well	25	14	11	1.469	0.225
Moderate/Poorly	92	39	53		
Depth of invasion					
1	8	7	1	10.512	0.015
2	4	3	1		
3	84	38	47		
4	20	5	15		
Lymph node metastasis					
0	33	26	7	24.989	<0.001
1	18	9	9		
2	25	9	16		
3	41	9	32		
Distance metastasis					
0	94	48	46	6.413	0.011
1	23	5	18		
Tumor-node-metastasis stage					
1	8	7	1	24.478	<0.001
2	37	26	11		
3	49	15	34		
4	23	5	18		
CEA level (μg/L)					
<5	104	48	56	0.139	0.709
≥ 5	10	4	6		

### High HSF1 expression predicted worse survival in patients with gastric cancer

**Fig. 3:**
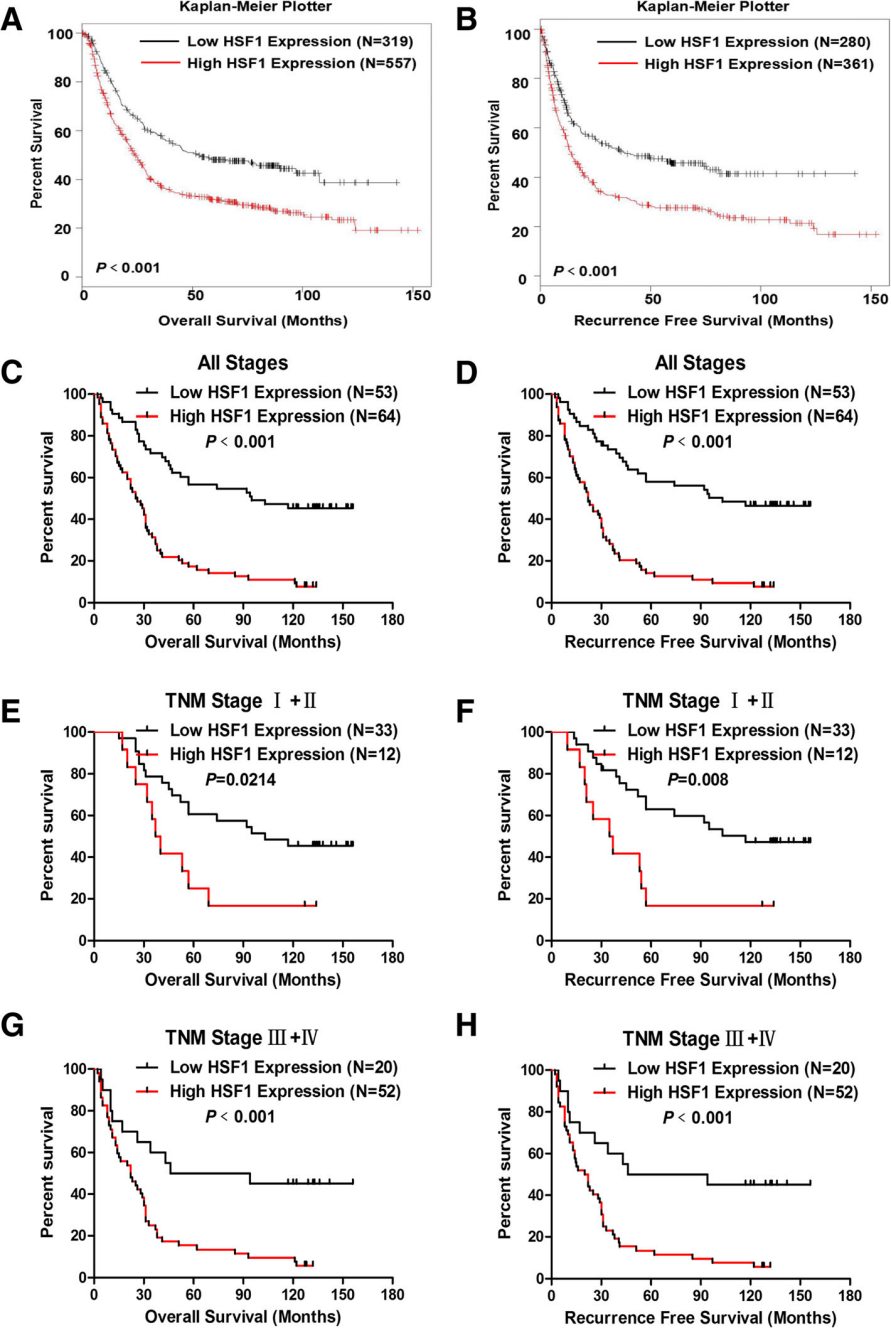
High HSF1 expression in tumours predicted poor prognosis in GC patients. (**a**&**b**) Overall survival (**a**, *P*<0.001) and recurrence-free survival (**b**, *P*<0.001) curves for the GC patient groups with low and high HSF1 mRNA levels in Kaplan-Meier Plotter. (**c**&**d**) Patients with higher HSF1 expression had worse overall survival (**c**, *P*<0.001) and recurrence-free survival (**d**, *P*<0.001) than those with lower HSF1 expression (*P*<0.001); (**e**&**f**) High HSF1 expression in GC tissues predicted worse overall survival (**e**, *P* = 0.0214) and recurrence-free survival (**f**, *P* = 0.008) in patients with early-stage GC (TNM stage: I and II);(**g**&**h**) High HSF1 expression in GC tissues predicted worse overall survival (**g**, *P*<0.001) and recurrence-free survival (**h**, *P*<0.001) in patients with advanced gastric cancer (TNM stage: III and IV)

To determine the prognostic value of HSF1 expression in GC patients, we first analysed the data in the Kaplan-Meier Plotter (http://kmplot.com). The results revealed that patients with high HSF1 level showed worse overall survival (OS, Fig. 3a, *P* <0.001) and recurrence-free survival (RFS, Fig. 3b, *P* <0.001). Second, we performed a Kaplan-Meier analysis to detect the prognostic role of HSF1 expression in our cohort. We found that patients with high HSF1 protein expression had poor OS (Fig. 3c, *P* <0.001) and RFS (Fig. 3d, *P* <0.001). Furthermore, we also determined the prognostic value of HSF1 expression in early (TNM stages I and II) and advanced (TNM stages III and IV) gastric cancer. The results indicated that patients with high HSF1 expression in GC tissues showed worse OS (Fig. 3e, *P* = 0.0214) and RFS (Fig. 3f, *P* = 0.008) than those with low HSF1 expression in early-stage GC; moreover, low HSF1 expression was associated with better OS (Fig. 3g, *P* <0.001) and RFS (Fig. 3h, *P* <0.001) than that observed in patients with high HSF1 expression in advanced GC.

Furthermore, univariate and multivariate analyses were performed to compare the impact of HSF1 expression and other clinicopathological factors on the prognosis of GC patients. Univariate analysis revealed that clinical variables including Bornmann classification (hazard ratio [HR] = 1.875; 95% confidence interval [CI]: 1.302–2.648; *P* = 0.001), depth of invasion (HR = 2.298, 95% CI: 1.537–3.434, *P*<0.001), lymph node metastasis (HR = 1.46, 95% CI: 1.215–1.756, *P*<0.001), distant metastasis (HR = 5.316, 95% CI: 3.129–9.032, *P*<0.001), tumour-node-metastasis stage (HR = 2.434, 95% CI: 1.8–3.29, *P*<0.001), tumour size (HR = 1.744, 95% CI: 1.146–2.654, *P* = 0.009) and HSF1 expression (HR = 3.274, 95% CI: 2.077–5.163, *P*<0.001) were significantly associated with OS. Furthermore, multivariate Cox regression analysis demonstrated HSF1 expression (HR = 2.598, 95% CI: 1.62–4.167, *P*<0.001) as an independent predictor of OS in GC patients (Table 2). Moreover, HSF1 expression was also an independent predictor of RFS (HR = 2.781, 95% CI: 1.721–4.494, *P*<0.001) in GC patients (Table 3). Taken together, these results indicated that high HSF1 expression was an independent prognostic factor for GC patients.

**Table 2:** Cox proportional-hazard regression analysis for Overall Survival

Characteristic	Univariate analysis				Multivariate analysis			
	*P* Value	HR	95.0% CI for Exp(B)		*P* Value	HR	95.0% CI for Exp(B)	
			Lower	Upper			Lower	Upper
Gender	0.138	1.384	0.901	2.124				
Age	0.472	0.854	0.556	1.313				
Tumor Location	0.106	0.827	0.657	1.041				
Tumor Size	0.009	1.744	1.146	2.654				
Bornmann classification	0.001	1.857	1.302	2.648				
Histologic type	0.608	0.861	0.486	1.525				
Differentiation	0.595	1.151	0.685	1.932				
Depth of invasion	<0.001	2.298	1.537	3.434	0.009	1.741	1.151	2.635
Lymph node metastasis	<0.001	1.46	1.215	1.756				
Distance metastasis	<0.001	5.316	3.129	9.032				
Tumor-node-metastasis stage	<0.001	2.434	1.8	3.29				
HSF1 Expression	<0.001	3.274	2.077	5.163	<0.001	2.598	1.62	4.167
CEA	0.873	1.065	0.492	2.308

**Table 3:** Cox proportional-hazard regression analysis for Recurrence Free Survival

Characteristic	Univariate analysis				Multivariate analysis			
	*P* Value	HR	95.0% CI for Exp(B)		*P* Value	HR	95.0% CI for Exp(B)	
			Lower	Upper			Lower	Upper
Gender	0.179	1.345	0.873	2.072				
Age	0.459	0.85	0.552	1.308				
Tumor Location	0.125	0.834	0.661	1.052				
Tumor Size	0.007	1.789	1.173	2.728				
Bornmann classification	0.001	1.889	1.315	2.713				
Histologic type	0.634	0.87	0.491	1.543				
Differentiation	0.612	1.144	0.681	1.921				
Depth of invasion	<0.001	2.217	1.494	3.291	0.013	1.679	1.118	2.523
Lymph node metastasis	<0.001	1.453	1.207	1.749				
Distance metastasis	<0.001	4.713	2.789	7.966				
Tumor-node-metastasis stage	<0.001	2.359	1.75	3.179				
HSF1 expression	<0.001	3.49	2.198	5.542	<0.001	2.781	1.721	4.494
CEA	0.905	1.048	0.484	2.273				

## Discussion

Our previous studies confirmed that HSP70/HSP90-organizing protein (HOP) could act as an oncogene by regulating malignant progression of gastric cancer, and high HOP expression predicted worse long-term outcome in GC patients [23, 25]. HSF1 is an upstream transcription factor of HOP [26]. Thus, in this study, we explore whether HSF1 expression is associated, similarly to that of HOP in GC, with an advanced tumour stage and poor prognosis. We show that HSF1 can be a potential biomarker to diagnose and evaluate the prognosis of GC patients, which is the first time the HSF1 expression has been associated with clinicopathological features and prognosis in GC. HSF1 is upregulated in GC tissues compared with non-tumour tissues in the TCGA cohort and FAHSYSU cohort. In addition, high HSF1 expression is significantly correlated with advanced GC malignant progression. Moreover, high HSF1 expression is associated with worse prognosis in patients undergoing surgery for GC. These findings suggest that *HSF1* may be involved in GC progression and may serve as a useful prognostic factor of patients with GC.

HSF1 is highly expressed in some types of solid cancers and may be used as a new biomarker to evaluate prognosis of cancer patients [12–19]. Fang et al. reported that the HSF1 level was predominantly elevated in HCC, especially in venous emboli from HCC, and the high HSF1 level was significantly correlated with multiple nodules, venous invasion, absence of capsular formation and high Edmondson-Steiner grade as well as poor outcome in HCC patients [27]. In breast cancer, a high HSF1 level was associated with histological grade, larger tumour size, and nodal involvement [14]. High expression of HSF1 protein is significantly associated with aggressive disease and poor survival in CRC, ESCC, ccRCC and osteosarcoma [12, 13, 16–18]. As shown in Fig. 2, HSF1 protein was localized in the nuclei of GC cells. However, interestingly, Zhang et al. demonstrated that HSF1 expression in peritumoural tissue, but not in HCC tissues, could serve as a sensitive biomarker for high-risk HCC early recurrence [28]. Moreover, HSF1 expression in stromal cells was associated with advanced tumour stage, lymph node metastasis and clinical stage, and poor outcome of ESCC [29]. In the present study, we have demonstrated that GC tissues present higher HSF1 expression levels than normal tissues, and high HSF1 level is associated with an advanced GC stage and worse long-term survival in early and advanced stages. However, the relationship between HSF1 expression in stromal cells and GC stage or patient’s survival may be explored in future studies.

In this study, we have found that high expression of HSF1 in GC tissues is related to more advanced depth of invasion, lymph node metastasis, distant metastasis and tumour-node-metastasis stage. Previous studies have reported that HSF1 could function as a potential tumour oncogene in different cancers. Mechanistically, a loss of AMPK activation amplified the HSF1 activity to promote the invasion and metastasis of pancreatic cancer [30]. In gastric cancer, HSF1 could promote GC cell proliferation, migration and invasion by directly interacting with MORC2 and binding to the enhancer of ArgBP2 [20]. HSF1 promotes the inhibition of epithelial-to-mesenchymal transition (EMT)-associated migration by low glucose by directly regulating Snail1 expression in HCC cells [31]. HSF1 could induce ovarian cancer cell EMT and promote cell migration and invasion [32]. We have also found that HSF1 could promote GC cell proliferation, migration and invasion. The upregulation of HSF1 in gastric cancer cells stimulates the expression of MMP2, MMP7 and MMP9. Moreover, HSF1 overexpression increases Vimentin, N-cadherin and Snail expression and decreases the level of E-cadherin (data not shown). Based on these studies and our results, we propose that HSF1 may be an oncogene regulating malignant progression of gastric cancer. Future studies are needed to elucidate the molecular mechanisms of HSF1 in GC.

## Conclusions

Taken together, our results indicate that high HSF1 expression indicates a poor prognosis in GC, and HSF1 can serve as an independent prognostic factor for the overall survival and recurrence-free survival in GC patients. Additional studies are required to clarify the molecular mechanisms by which HSF1 promotes GC development.

## Abbreviations

HSF1: Heat shock factor 1
IHC: Immunohistochemical
OS: Overall survival
RFS: Recurrence-free survival
